# Parkinson's Disease in a Dish: What Patient Specific-Reprogrammed Somatic Cells Can Tell Us about Parkinson's Disease, If Anything?

**DOI:** 10.1155/2012/926147

**Published:** 2012-12-20

**Authors:** J. Drouin-Ouellet, R. A. Barker

**Affiliations:** ^1^Neuroscience Axis, CHUL Research Center (CHUQ), T2-50, 2705 Boul. Laurier, Quebec City, QC, Canada G1V 4G2; ^2^Department of Clinical Neurosciences, Cambridge Centre for Brain Repair, University of Cambridge, Cambridge CB2 0PY, UK

## Abstract

Technologies allowing for the derivation of patient-specific neurons from somatic cells are emerging as powerful *in vitro* tools to investigate the intrinsic cellular pathological behaviours of the diseases that affect these patients. While the use of patient-derived neurons to model Parkinson's disease (PD) has only just begun, these approaches have allowed us to begin investigating disease pathogenesis in a unique way. In this paper, we discuss the advances made in the field of cellular reprogramming to model PD and discuss the pros and cons associated with the use of such cells.

## 1. Introduction

 Parkinson's disease (PD) is a neurodegenerative disorder for which there is currently no disease modifying treatment but a number of symptomatic therapies. The disease is clinically defined by its motor features, which include rigidity, bradykinesia, resting tremor, and postural and gait disturbances. PD patients are also affected by a variety of nonmotor deficits such as hyposmia, autonomic dysfunction, sleep disturbances, cognitive impairment, and psychiatric symptoms [1]. The core pathology is the loss of dopaminergic neurons of the substantia nigra, although many other neuronal populations are also affected including the noradrenergic locus coeruleus, serotoninergic raphe nucleus, and cholinergic basal forebrain systems as well as range of other structures such as the cortex, olfactory bulb, and even the enteric nervous system. Whilst the loss of the dopaminergic nigrostriatal pathway is the classical biochemical deficit in PD, pathologically it is the formation of Lewy bodies. Lewy bodies are composed of insoluble protein aggregates mainly made up of the protein *α*-synuclein (*α*-syn). 

While this summary is a useful starting point in our understanding of PD, it is only an approximation of the true nature of this disorder as the disease is now recognised to be very heterogeneous at both the clinical and pathological levels [2]. Therefore, better classifying subtypes of PD will be necessary as will be the need to understand the differences in disease pathogenesis at the cellular and systems levels in these different forms of PD.

One approach, which holds great promise in terms of dissecting the different cellular events underlying the genesis of different subtypes of disease, is the area of cellular reprogramming of somatic cells from PD individuals themselves. Following the seminal demonstration that differentiated human somatic cells could be reprogrammed into a pluripotent state by the overexpression of a set of defined transcription factors (Oct4, Sox2 with either the combinations of Klf4 and Myc or Nanog, and Lin28) [3–5], the door to a whole new field of research has been opened. This technology using induced pluripotent stem (iPS) cells allows for cells to be grown from patients themselves which have

1. the capacity to proliferate indefinitely in culture;
2. a pluripotency profile, such that they can in theory be differentiated into any cell type, including neurons and more specifically dopaminergic nerve cells. iPS cells thus hold great promise for the *in vitro* modeling of neurodegenerative disorders, including PD.

More recently, it has been shown that one can actually directly reprogram human somatic cells into neurons, thereby avoiding the pluripotent state. This is also another emerging alternative approach to study patient-specific pathological disease processes. These reprogrammed cells could also be extremely useful for screening potential compounds for therapeutic purposes. 

Since the first reports on human iPS cells and induced neurons (iN) were published, the field has moved forward at great speed, and in this paper, we present the highlights of the field as well as where it is going in terms of therapeutic applications.

## 2. Modeling PD with iPS Cells

iPS cells derived from patients offer a powerful *in vitro* model to study disease as these cells carry the necessary genetic risk factors for that disorder. The first iPS cells generated from PD patients were derived from fibroblasts harvested from skin biopsies and provided the first opportunity to truly study human pathological processes and drug development *in vitro* [6]. 

One of the first studies to successfully produce midbrain dopaminergic neurons from mouse iPS cells obtained neurons that expressed a number of markers specific to the midbrain dopaminergic system such as Nurr1, Pitx3, and tyrosine hydroxylase (TH) and had an electrophysiological pattern characteristic of nigral dopaminergic neurons [7]. Subsequently, human iPS cells were also efficiently differentiated into committed neural stem cells and dopaminergic neurons (iDA) using identical differentiation protocols to those used for human embryonic stem (ES) cells. Using such protocols, 30% TH differentiation was achieved, and the functionality of these neurons was further demonstrated by iDA survival with improvements in behavioral deficits when they were grafted into 6-hydroxydopamine- (6-OHDA-) lesioned rats [8]. Other groups have now further refined the differentiation protocol to obtain a more reliable and efficient production of iDA by either modulating FGF/ERK signaling [9] or by genetic engineering iPS cells with lentiviral vectors regulating the expression of Lmx1a [10]. 

At least 10 subtypes of dopaminergic neurons exist in the adult brain (A8–A17) [23], all of which show specific electrophysiological, neurochemical, and transcriptional profiles [24–26]. Ventral midbrain dopaminergic neurons—especially those found in the substantia nigra—are those most vulnerable to degeneration in PD [27, 28], and it is known that these A9 nigral dopaminergic neurons are needed in cell transplants to restore function in animal models of PD [12, 29–31]. If we hope to use iN to model PD, it is imperative that these neurons are genuine human midbrain nigral neurons. With this in mind, a human iPS cell differentiation protocol targeting both early dorsalizing and ventralizing neural patterning pathways has been proposed [12]. Using this protocol, differentiated cells expressed all of the relevant markers, although electrophysiological studies were not performed which limits what one can say about these cells. 

Several groups have now been able to produce PD patient-specific iDA derived from iPS cells. The first of these used fibroblasts from idiopathic PD patient and showed that iDA derived from iPS cells, regardless of the underlying disease or the age of the donor, did not show any disease-related phenotype [11]. This particular study also highlighted that residual transgene expression in virus-carrying iPS cells influenced their molecular properties and recommended that derivation methods free of reprogramming factors should be used. 

This led to the first proof of concept study in which iDA derived from iPS cells from sporadic and LRRK2-associated PD patients displayed distinct disease-specific pathology [13]. In contrast, Soldner and colleagues [11] found no differences between the iDA from PD patients and controls in any measure after 30 days in culture. In their study, the majority of iN expressed the ventral midbrain dopaminergic neuronal phenotype, as compared to only 10% in the previous reports [11, 13]. However, time *in vitro* may have been a more critical factor here, as the long-term culture (<75 days) of iDA derived from sporadic PD cases revealed an altered morphology in PD-iDA, in particular, a decrease in the number and length of neurites and an increased susceptibility to degeneration and defective autophagosome clearance [13]. 

While the vast majority of PD cases are idiopathic (>85%), several causative genes have been identified in families displaying Mendelian inheritance of the disorder [32]. As a result, one obvious application of iDA is to study dopaminergic neuronal behavior associated with these genetic mutations. Thus far, four PD-related genes have been studied using iPS cell technology: *SNCA*, *Leucine-rich repeat kinase 2* (*LRRK2*), phosphatase and tensin homolog (*PTEN*)*-*induced putative kinase 1 (*PINK1*), and *Parkin* (Table 1). 

### 2.1. SNCA

Alpha *SNCA* is the gene coding for *α*-syn, the main component of Lewy bodies [33]. It was also the first gene with mutations identified to cause autosomal dominant PD. Thus far, four different missense mutations (A53T, A30P, E46 K, and H50Q) have been linked to familial PD, as well as duplications and triplications of the entire gene [34, 35]. So far, iPS cell lines from patients carrying a triplication of SNCA have been generated and differentiated into iDA [16]. While the fibroblasts did not express *α*-syn, a two fold increase in *α*-syn mRNA and protein expression was reported in patient-derived iDA when compared to cells from unaffected first-degree relatives sharing a similar genetic background. This provides the first proof-of-principle that this type of approach is viable to study Mendelian genetically driven pathological processes involved in *α*-synucleinopathies. However, given the significant variability of clonal variation and efficiency of differentiation, this study also highlights the importance of generating multiple iPS cell lines from a single individual to identify clones with the capacity to differentiate into cell type of interest and also to compare neuronal cultures with equivalent differentiation efficiency [35].

 A subsequent study to differentiate iPS cell lines into neurons from patients carrying a triplication of *SNCA* has further reported an accumulation of *α*-syn, overexpression of markers of oxidative stress, and increased sensitivity to peroxide-induced oxidative stress [15]. These findings suggest that these features are, at least in part, cell autonomous and that this approach with iPS cells can be a valuable way to study pathophysiological processes in the relevant cells from patients with specific genetic abnormalities. In this study, both TH-positive and TH-negative neurons exhibited ubiquitinated intracellular inclusions [15], indicating that patient-derived iPS cells with *SNCA* triplication can also be used to investigate selective vulnerabilities across neuronal subtypes associated with overexpression of wild-type *α*-syn.

One of the main considerations in modelling diseases by iPS cells *in vitro* includes the difficulty in distinguishing subtle disease-relevant phenotypic changes and how relevant these are to what takes place in the patients aging central nervous system (CNS). The lack of genetically matched controls combined with the high variability of the biological characteristics of the cells and cell lines derived from a single healthy donor are also important hurtles [11, 36, 37]. In an attempt to overcome these obstacles, Soldner and colleagues (2011) generated human iPS cell lines from patients carrying the A53T (G209) mutation in the *SNCA* gene which they then corrected using zinc finger nuclease-mediated genome editing [17]. They further confirmed the loss of expression of the A53T mutated transcript and demonstrated that this genetic repair did not compromise the ability to differentiate into iDA. By so doing, they generated iDA that differed only in this gene (i.e., a gene which gives the cell its susceptibility for PD), providing genetically matched control cells to study the effects of that specific mutation. However, while this approach is appealing to study cellular mechanisms associated with Mendelian forms of PD, it excludes the use of iPS cell lines to investigate idiopathic PD as such mutations do not exist.

Intracellular protein interactions relevant to PD have also been tackled using iDA reprogrammed from iPS cells derived from patients carrying a mutation in the *glucocerebrosidase* gene. This mutation is known to alter sphingolipid metabolism and has also been linked to parkinsonism [38]. Indeed, it has been suggested that there is a bidirectional effect of *α*-syn and *glucocerebrosidase* which acts to form a positive feedback loop that leads to a self-propagating disease. Namely, the *glucocerebrosidase* mutations jeopardize lysosomal protein degradation leading to aggregation of *α*-syn and neurotoxicity. Conversely, in iDA with wild-type *glucocerebrosidase*, *α*-syn inhibits lysosomal activity, suggesting that a loss of *glucocerebrosidase* in some patients with PD and a *glucocerebrosidase* heterozygote mutation could catalyse *α*-syn aggregation and by so doing contribute to the pathogenesis of their PD [22]. 

### 2.2. LRRK2

Mutations in the *LRRK2* gene have been reported to be the most frequent cause of late-onset autosomal dominant, as well as sporadic, PD [39, 40]. However, these mutations give rise to inconsistent pathological features, ranging from Lewy body inclusions to a strictly nigral degeneration with an absence of Lewy bodies [41]. LRRK2 is a kinase with many domains which is capable of controlling many protein-protein interactions. While it is thought that changes in LRRK2 protein domains can influence kinase activity by interfering with other proteins [42], the mechanism underlying the pathogenesis of LRRK2-PD patients is currently unknown. Studies of iPS cells derived from a single patient carrying the G2019S mutation in the *LRRK2* gene demonstrated increased accumulation of *α*-syn, an upregulation of key oxidative stress response genes and a selective vulnerability of TH-positive iN to neurotoxins, including H_2_O_2_, MG-132, and 6-OHDA [18]. As for iDA derived from sporadic PD cases, Sánchez-Danés and colleagues (2012) have reported that long-term cultures of iDA carrying a *LRRK2* mutation displayed abnormal morphology, defective autophagosome clearance, and increased susceptibility to degeneration. Consistent with previous findings, aberrant diffuse cytoplasmic accumulation of *α*-syn in iDA differentiated from these LRRK2-PD iPS cells was also observed when compared to both control and idiopathic PD-derived iPS cells [13]. Furthermore, iN derived from patients carrying either the homozygous G2019S or the heterozygous R1441C show that while the mitochondrial electron transport chain is intact, these cells exhibit a neuronal cell type-specific increased sensitivity to mitochondria chemical stressors that depolarize mitochondria using K^+^ ions but not protons (H^+^) [19]. This suggests that LRRK2 is involved in the cell ability to respond to mitochondria damaged by the influx of K^+^ ions.

### 2.3. Parkin and PINK1

Mutations in the *parkin* gene are a cause of autosomal recessive PD, which usually manifests early in life. Indeed, homozygous mutations in *parkin* are the most frequent causes of juvenile PD. This early onset form of the disease is characterized by nigral neuronal loss and gliosis but rarely has Lewy bodies. The Parkin protein functions as an E3 ubiquitin ligase to conjugate ubiquitin proteins to lysine residues of target proteins [43]. 

PINK1 is a mitochondrial kinase for which pathogenic mutations are the second most common cause of autosomal recessive early onset PD [44]. Loss-of-function mutations in the gene are thought to either compromise the kinase activity of PINK1 or interfere with its protein stability [45, 46]. 

Both Parkin and PINK1 are involved in mitochondrial function [47], and PINK1 has been suggested to function upstream of Parkin [48–50]. Midbrain iDA reprogrammed from iPS cells derived from skin fibroblasts of PD patients with *parkin* mutation exhibit increased spontaneous dopamine release and uptake, as well as an elevation in reactive oxygen species production. Of note, lentiviral expression of *parkin*, but not its PD-linked mutant, rescues these phenotypes. However, mitochondria are not seemingly affected in these cells and the levels of TH and *α*-syn expression are not different when compared to cells from control subjects. [20]. In contrast, a further study showed that in human iDA carrying an endogenous *PINK1* mutation, Parkin did not translocate to mitochondria, suggesting that mutations in PINK1 result in diminished recruitment of Parkin. This impairment was abrogated by the expression of normal PINK1. In addition, as opposed to wild-type iDA, mitochondrial DNA did not decrease in depolarized mutant PINK1 iDA [21].

As it has been demonstrated for *LRRK2* mutations, iN cells carrying a recessive homozygous Q456X *PINK1* mutation show an impaired capacity to respond to mitochondria damaged by the influx of K^+^ ions. These cells are also less able to respond to oxidative stress. Vulnerability to chemical stressors could be rescued by the antioxidant coenzyme Q_10_ and a LRRK2 inhibitor, but not rapamcyin [19]. This suggests that the increased production of ROS induced by the loss of PINK1 could be associated with LRRK2 functions. 

While a relatively small amount of studies employing iPS cells from PD patients harboring a genetic mutation have been performed, they have been instrumental in clarifying some of the roles of critical proteins underlying familial forms of PD and their interactions with one another. These studies can further provide clues on how to tackle cell-autonomous pathological mechanisms in relevant neurons from idiopathic PD patients who represent the vast majority of cases. However, they are not able to investigate how different cellular compartments (e.g., glial cells) may interact in the overall disease process.

## 3. Modelling PD with Direct Neuronal Conversion 

In recent years, neurons differentiated from iPS cells have provided new insights into the cellular mechanisms involved in the pathophysiology of PD. However, concerns remain with respect to their utility to do this given that they are reprogrammed back to a more pluripotent stage. To overcome this issue, several groups have developed methods that allow direct conversion of human differentiated somatic cells, such as fibroblasts, into functional neurons avoiding a pluripotent state.

The first proof-of-concept study was achieved by the conversion of mouse embryonic and postnatal fibroblasts into functional neurons by the overexpression of three transcription factors (Ascl1, Brn2, and Mytl1). These iN displayed neuronal properties such as the generation of action potentials as well as synapse formation [51]. Human fibroblasts have also been successfully converted into functional neurons by overexpressing the same transcription factors [52]. Several subsequent studies have been undertaken with the aim of optimizing these conversion methods. For example, functional neurons have been generated using two of the aftermentioned factors (Brn2 and Mytl1) with the addition of a microRNA (miR-124) [53] or with the combination of microRNAs (miR-124 and miR-9/9*) and NeuroD2 [54]. While these methods generated cells that exhibited both electrophysiological and morphological characteristics typical of neurons, their neuronal subtype identity remained unclear.

The ability to convert human fibroblasts into functional glutamatergic forebrain neurons has been shown with cells from Alzheimer's disease patients, using a combination of four transcription factors (Ascl1, Brn2, Mytl1, and Zic1) [55]. The addition of two further transcription factors specific for the dopaminergic lineage (Lmx1a and FoxA2) is also sufficient to generate cells expressing TH and a dopaminergic morphology with a 10% conversion efficiency [52]. Furthermore, the reduction of this combination of transcription factors to only three (Ascl1, Nurr1, and Lmx1a) was sufficient to obtain cells with a dopaminergic neuronal-like morphology and appropriate electrophysiological properties [14]. However, the gene expression profiles of these reprogrammed DA neurons differed significantly from primary midbrain DA neurons in these studies, and so more recent attempts to generate iDA-like midbrain dopaminergic neurons have used six reprogramming factors (Ascl1, Pitx3, Nurr1, Lmx1a, Foxa2, and En1), as well as the patterning factors Shh and FGF8 [56]. The iDA so generated expressed the generic dopaminergic markers TH, dopamine transporter (DAT), aromatic L-amino acid decarboxylase (AADC), and vesicular monoamine transporter 2 (VMAT2) and were also shown to release dopamine but only partially restored dopamine system functions *in vivo *in animal models of PD. These iDA cells also failed to show similar levels of the relevant transcription factors when compared to embryonic or adult midbrain dopamine neurons [56]. More recently, a combination of five transcription factors (Ascl1, Pitx3, Nurr1, Sox2, and Ngn2) generated iDA that were able to better reverse deficits when grafted into the 6-OHDA rat model of PD, suggesting that these reprogrammed cells display more properties of functional midbrain dopaminergic neurons [57].

Because the direct conversion does not go through a proliferative state, the quantity of neurons that can be obtained is limited by the accessible number of fibroblasts used as starting material for conversion. Nevertheless, direct conversion of patient's fibroblasts into relevant neuronal subtypes is very promising for disease modeling as well as potentially being useful for autologous cell therapy. 

## 4. iDA as a Cell Therapy Source for Grafting in PD 

Cell therapy is one of the promising experimental therapeutic approaches currently being tested in the clinic in patients with PD. However, ethical and logistical issues associated with the use of human fetal tissue prevent the widespread adoption of these cells in the clinical setting. The use of ES cells has been proposed as an alternative, mainly because these cells have the potential to generate all type of cells and provide an unlimited source of donor tissue [58]. However, the use of these cells has been hampered by (i) ethical issues, (ii) their tumourigenic potential; (iii) their ability to generate sufficient numbers of true nigral dopaminergic neurons; (iv) the possible immune rejection of them due to host-donor immunological incompatibilities. Thus efforts have been made towards developing protocols that result in large numbers of nigral dopaminergic neurons, in the absence of any proliferative cell type or immune reaction to them. 

iDA generated from iPS cells derived from the skin fibroblasts of patients are very appealing in this respect. Indeed, do these cells circumvent not only ethical issues associated with the use of fetal tissue, but also the risk of immune rejection. One other benefit in using iPS cells is the possibility of rejuvenating the cells from an aged patient and thus eliminating the pathologies associated with aging including the risk of Lewy body pathology in the transplant. This potential for iDA derived from iPS cells to be used as a cell replacement therapy has been assessed in the 6-OHDA rat model of PD. Studies have shown that grafted iPS cells functionally integrate into the host brain, and a large proportion of them differentiate into dopaminergic neurons expressing relevant markers such as TH, En1, VMAT2, and DAT four weeks following grafting [7]. While the vast majority of grafted animals showed behavioral improvements [7], continuous proliferation of transplanted cells, reminiscent of teratomas, was observed in some cases, and this raises serious safety concerns with their use [7, 59]. Differentiated iN and iDA have also been grafted in the 6-OHDA lesioned rat. Here, it has been shown that a small proportion of iN send out nondopaminergic connections into the surrounding white matter and that the iDA themselves had TH+ fibers that projected more within the graft than the host striatum [60]. These grafted cells did though provide a degree of functional recovery in amphetamine and apomorphine-induced rotational asymmetry in the majority of animals but failed to show improvement in behavioral tests that rely more on the connectivity of iDA with the host striatum, such as the cylinder test and the adjustment stepping test [60]. 

Moreover, several subpopulations of iDA were found within the graft, including a small number of forebrain- and hippocampal-like DA neurons. Transplantation of differentiated cells did not, though, generate teratomas. 

Directly converted iDA from fibroblasts have also been transplanted into 6-OHDA lesioned rodents [56, 57]. Grafted mouse midbrain-like iDA, reprogrammed from tail tip fibroblasts, maintained their neuronal morphology, extended TH+ fibers into the host striatum, generated a rise in local dopamine levels and improved amphetamine-induced rotational behavior two months posttransplantation [56]. Human iDA also retained their dopaminergic neuron-like properties *in vivo *for up to 4 months post-transplantation [57].

Several proof-of-concept studies have thus been undertaken to show the capacity of iPS cells and iDA to provide functional recovery in toxin-induced animal models of PD. However, a number of questions remain to be answered such as the following.

Do these cells really form mature nigral dopaminergic neurons with their characteristic axonal outgrowth and arborisation?

How safe are these cells when used in this way? 

Do these cells retain disease-specific vulnerability that will adversely affect their long-term beneficial effects? 

In the case of Mendelian forms of PD, can we repair disease-related mutations *in vitro* and transplant corrected cells?

Can these cells be reprogrammed to replace all the cell losses seen in this disease?

## 5. Advantages and Limitations of the Use of Fibroblast-Derived Cells to Mimic PD Pathology 

iPS cells have enormous potential for better understanding and treating PD but they also present a number of problems, some of which, but perhaps not all, will be resolved as the technology evolves. The clear advantage of using iDA derived from skin fibroblasts is that these cells are patient-specific, primary human cells which are easily available and relatively easy to culture. These cells thus provide multiple possibilities in the field of personalized medicine, with the potential for drug screening/testing on a range of affected neuronal cell populations.

There are also concerns with the use of iPS cell technology to study age-related pathologies such as PD. The induction of pluripotency is accompanied with a progressive elongation of telomeres with passaging [61–64], thus rejuvenating the cells in a similar way to the embryonic stem cell state, even in cells derived from aged individuals. However, the telomere chromatin returns to a mature state, similar to the one they were originally in when harvested, when differentiated again. iPS cells also retain DNA methylation patterns that are indicative of their original state before derivation [65–67]. The use of differentiated cells derived from iPS cells to model PD can be hampered by aberrant chromatin formation at the telomere level or elsewhere, making them more predisposed to telomere shortening and/or malignant transformation [68]. Going through the iPS cell stage creates reprogrammed iN that could be too young to exhibit PD phenotypes, and it has been suggested that it may therefore be necessary to accelerate PD-related phenotypes with exogenous stressors such as *in vitro* exposure to oxidative stress, neurotoxins, or overexpression of PD-associated proteins [11]. In this respect, longer culture times maybe all that is necessary, and indeed it has been shown that with iDA, the expression of these phenotypes does occur with increasing time in culture [13]. 

One other challenge that has to be overcome when modelling diseases using somatic cells as a primary source is the variable biological characteristics of cells from healthy donors as controls. Aspects that can vary include differences in genetic background, as well as in the cell derivation and differentiation processes [11, 36, 37], along with the genetic alterations introduced during the reprogramming process [69, 70]. Finally, whether human iPS cells are ever truly equivalent to the cell type into which they are being reprogrammed at the molecular and functional levels is another question that still needs to be answered [71].

Several major obstacles still remain to be overcome before the use of iPS therapy can be routinely performed in the clinic. Safety is a large concern, as these cells should be free from genetic aberrations and capable of differentiating into fully committed cells. A better understanding of the gene profiling that guides their development and differentiation would allow for the development of newer, safer techniques for human iPS cell derivation. Moreover, understanding the best approach to evaluate the properties of human iPS cell-derived differentiated cells and compare them with their natural counterparts *in vivo* will be critical in this regard. While exogenously used factors can be turned off during and after reprogramming, the possibility that they could either be turned back on during differentiation or that they could integrate and aberrantly activate oncogenes still exists. Although much work is therefore needed to optimize reprogramming methods and ensure a safe and efficient means of neuronal derivation, as well as their detailed cellular behavior, researchers have been increasingly hopeful that iPS cells or iDA will be a source of cells for studying disease pathogenesis as well as possibly cell replacement therapy in PD. 

## 6. Somatic Cells for Future Alternative PD Modelling 

PD is a complex disorder involving multiple systems and various cell types, including both neurons and glia. The ability to model PD using the conversion of somatic cells from PD patients would therefore benefit from the development of cocultures of multiple induced cell lineages. Aside from iDA, successful differentiation into neuronal subtypes includes glutamatergic neurons [72–74], GABAergic neurons [75], and motorneurons [76, 77]. Differentiation into an astrocyte lineage has been demonstrated with human iPS cells [78–83], although these cells represent only a small proportion of the total neural cells in culture. A recent study has induced neural stem cells from mouse fibroblasts using a shorter version of the reprogramming protocol, and these cells retained the capacity to differentiate into the three main neural lineages neurons, astrocytes, and oligodendrocytes [84]. Human iPS cells derived from fetal fibroblasts can also be differentiated into endothelial cells and recapitulate angiogenesis both *in vitro* and *in vivo *[85]. While microglia have been generated from mouse embryonic stem cells [86] and lineage-negative bone marrow cells from adult mice [87], successful reprogramming of somatic cells, such as fibroblasts into this cell type, has yet to be reported.

Studying the cellular pathology of different cell types could unravel their role in disease processes and by so doing help in the development of better therapeutic approaches. For example, in addition to iN reprogrammed from iPS cells derived from Huntington's disease patients that display changes in electrophysiology, metabolism, cell adhesion, and cell vulnerability to stressors [88], induced astrocytes exhibit cytoplasmic empty vacuoles [89], an abnormal phenotype that has been observed in peripheral blood lymphoblasts harvested from Huntington's disease patients [90]. While the role of these vacuoles is not known, these findings underline the importance of assessing disease-specific phenotypes within each cell type involved in the pathophysiology of the disease. Furthermore, the establishment of an *in vitro* system in which multiple types of cells affected in PD could coexist would clearly provide additional information as to how they interact within the PD brain to generate the disease state. 

## 7. Conclusions

Because iPS cells and directly converted cells from fibroblasts circumvent many of the ethical considerations that surround the use of ES cells, as well as originating from the patient themselves, they offer a wide range of possibilities for mainstream clinical use across the globe. One promising therapeutic application would be to generate iPS cells from patients that have genetic diseases, repair the genetic defect, differentiate the cells into the desired phenotype, and then reintroduce them into the patient. These cells could also be helpful *in vitro* in identifying new pathogenic pathways as well as novel biomarkers for individuals at risk of developing PD or complications of it (e.g., PD dementia). While a tremendous amount of work is still needed to reach these goals, the derivation of iPS cells from both familial and idiopathic PD patients to model the disease *in vitro* uniquely allows for therapeutic cell manipulations that cannot be performed *in vivo*. This will ultimately define in different subgroups of patients whether a given mutation or more complex gene interaction provides a cellular vulnerability or whether the disease process requires additional environmental or epigenetic factors. Although still in its infancy, the diversity of applications associated with the use of iPS and directly converted cells from fibroblasts makes this field of research one of the most promising for the *in vitro* modeling of PD and through this, the derivation of truly novel therapeutic approaches. 

## Figures and Tables

**Table 1:** Summary of studies that have used dermal fibroblasts from PD patients to model the disease.

Forms of PD	Source of cells	Main findings	References
Sporadic			
	Dermal fibroblasts ↓	PD-specific iPS cells are able to generate dopaminergic neurons	[11]
	iPSC	New human iPS cell differentiation protocol to produce vmDA neuron	[12]
	↓ iDA	Morphological alterations (reduced numbers of neuritis and neurite arborization), accumulation of autophagic vacuoles	[13]

	Dermal fibroblasts ↓ iDA	Rapid and efficient induction of iDA from human PD patient fibroblasts	[14]

Familial			
SNCA triplication	Dermal fibroblasts ↓	Accumulation of *α*-syn, inherent overexpression of markers of oxidative stress, and sensitivity to peroxide induced oxidative stress	[15]
SNCA triplication	iPSC	Production of double the amount of *α*-syn as neurons from the unaffected relative	[16]
SNCA A53T mutation	↓ iN/iDA	Successful genetic repair of the mutation	[17]

LRRK2 G2019S mutation	Dermal fibroblasts ↓	Increased expression of key oxidative stress-response genes and *α*-syn protein. Increased sensitivity to caspase-3 activation and cell death caused by exposure to stress agents	[18]
LRRK2 G2019S mutation	iPSC	Morphological alterations (reduced numbers of neurites and neurite arborization), accumulation of autophagic vacuoles	[13]
LRRK2 G2019S, R1441C mutations	↓ iDA	Vulnerability associated with mitochondrial dysfunction which could be rescued with coenzyme Q10, rapamycin, and the LRRK2 inhibitor GW5074	[19]

Parkin mutation		Increased transcription of monoamine oxidases and oxidative stress, reduced DA uptake and increased spontaneous DA release	[20]
PINK1 mutation	Dermal fibroblasts ↓ iPSC iDA	Impaired recruitment to lentivirally expressed parkin to mitochondria, increased mitochondria copy number, upregulation of PGC-1*α*; corrected by lentiviral expression of wild-type PINK1	[21]
PINK1 Q456X mutation		Vulnerability associated with mitochondrial dysfunction which could be rescued with coenzyme Q10, rapamycin, and the LRRK2 inhibitor GW5074	[19]

Risk gene			
Glucocerebrosidase	Dermal fibroblasts ↓ iPSC ↓ iDA	Dramatic increase in *α*-syn protein levels with accumulation of *α*-syn, which results in neurotoxicity through aggregation dependent mechanisms	[22]

Abbreviations: *α*-syn: *α*-synuclein; DA: dopamine; iDA: induced dopaminergic neurons; iPS: induced pluripotent stem; LRRK2: Leucine-rich repeat kinase 2; PD: Parkinson's disease; PGC-1*α* Peroxisome proliferator-activated receptor-*γ* coactivator 1*α*; SNCA: *α*-synuclein gene; vmDA: ventral mesencephalon dopaminergic.
